# Allicin mitigates fumonisin B_1_-induced kidney toxicity in quails: Modulating fibrosis, NF-κB signaling pathway, and mitochondrial damage

**DOI:** 10.1016/j.psj.2025.105356

**Published:** 2025-05-28

**Authors:** Yangwan Zhang, Yihao He, Xueyan Zhu, Yang Liu, Changyu Cao

**Affiliations:** a College of Animal Science and Technology, Foshan University, Foshan Guangdong 528225, China; b Quality Control Technical Center (Foshan) of National Famous and Special Agricultural Products (CAQS-GAP-KZZX043), College of Food Science and Engineering, Foshan University, Foshan Guangdong 528225, China; c Foshan University Veterinary Teaching Hospital, Foshan, Guangdong 528231, China

**Keywords:** FB_1_, Allicin, Quail, Nephrotoxicity

## Abstract

Fumonisin B_1_ (FB_1_) is a common mycotoxin, which is a water-soluble metabolite produced through the metabolism of Fusarium verticillioides and Fusarium proliferator. Crops and feedstuffs are widely contaminated by FB_1_ from the environment, posing risks to livestock and human health. Currently, there is no therapeutic approach available for FB_1_ intoxication. Allicin, an organic sulfur compound extracted from garlic, exhibits anti-inflammatory, antioxidant, antibacterial, hepatoprotective, cardioprotective, and intestinal regulatory properties. However, investigations into allicin’s role in alleviating FB_1_-induced quail nephrotoxicity remain relatively limited. This study thus aimed to elucidate the mechanisms by which allicin exerts a protective effect against FB_1_-induced kidney injury in juvenile quails. A total of 150 juvenile quails were randomly divided into five groups and treated with varying allicin concentrations (0, 50, 100, 500, and 1000 mg/kg) for 8 weeks. The blood and kidney tissues were subsequently screened using serum biochemical indices and histological staining, which suggested that 500 mg/kg of allicin was the optimal concentration that exerts protection to the quail kidneys. Another 120 juvenile quail were randomly divided into four groups (*n* = 30): control, allicin, FB_1_, and allicin+FB_1_. The pathological changes in kidney tissues induced by FB_1_ and genes associated with nuclear xenobiotic receptors (NXRs), inflammation, fibrosis, and mitochondrial damage were evaluated after 8 weeks. FB_1_ triggered kidney fibrosis and mitochondrial injury by activating the NF-κB signaling pathway, modulating NXR expression, and regulating corresponding CYP450 subtypes, which culminated in pathological injury to kidney tissues. Notably, allicin alleviated FB_1_-induced kidney injury in quails, possibly by inhibiting the NF-κB pathway, fibrosis, and mitochondrial damage, suggesting the potential application of allicin in preventing FB_1_-induced toxicity in quail.

## Introduction

Mycotoxins are secondary metabolites produced by various fungi during metabolic processes. They harbor potential toxicity to animals (Gao et al., 2023). For instance, grain crops infected by Fusarium are a major source of FB_1_ enrichment, which poses a threat to animal and human health by inducing various diseases, such as esophageal cancer (Alizadeh et al., 2012),neural tube defects (Lumsangkul et al., 2021),mycotoxicosis (Chen et al., 2021),equine leukoencephalomalacia (Awuchi et al., 2022),nephrotoxicity,immunotoxicity (Sun et al., 2022; Yang et al., 2020),liver damage (Papatsiros et al., 2021). The International Agency for Research on Cancer has classified FB_1_ as a designated potential human carcinogen. The International Agency for Research on Cancer classifies FB_1_ as a designated potential human carcinogen, which makes FB_1_ a serious mycotoxin pollution that requires synergy from all the relevant sectors to contain (Gao et al., 2023).

Allicin is an organic sulfur-containing compound extracted from garlic. It has multiple biological functions, such as anti-inflammatory, antioxidant, antibacterial, anti-cancer, hepatoprotective, cardiovascular, and intestinal regulation (Catanzaro et al., 2022; Mocayar Marón et al., 2020). Studies postulate that allicin has a significant protective effect on renal injury induced by gentamicin, acrylamide, and ischemia/reperfusion (Li et al., 2023; Nabavi et al., 2012; Xu et al., 2023). The specific mechanism potentially involves apoptosis, oxidative stress, inflammatory reaction, fibrosis, and other molecular and cellular pathways (Arellano-Buendía et al., 2020; El-Kashef et al., 2015; Li et al., 2023; Shan et al., 2021). Allicin also exerts a protective effect on aflatoxin mutagenicity and liver carcinogenicity (Rauf et al., 2022; Sheen et al., 2001). Moreover, it plays an important role in maintaining mitochondrial homeostasis, repairing mitochondrial dysfunction, alleviating fibrosis, and reducing inflammatory response (Abdel-Daim et al., 2019; Liu et al., 2015). However, the potential and mechanism of allicin to alleviate renal toxicity caused by FB_1_ remains largely unknown.

The kidneys are second to the heart in oxygen consumption and mitochondrial quantity (Di Paola et al., 2022). The abundant blood perfusion and high structural and physiological complexity of the kidneys make them highly susceptible to chemical toxins and various types of damage (Barnett and Cummings, 2019; Edwards and Kurtcuoglu, 2022; Yadav et al., 2024). FB_1_ can be metabolized and accumulated in the kidney, causing oxidative and mitochondrial damage and inflammatory reactions (Li et al., 2023; Shan et al., 2021). Yayeh T (Yayeh et al., 2021) reported that FB_1_ induced apoptosis and necrosis of mouse kidney cells. Aldawood N (Aldawood et al., 2023) reported that early exposure to FB_1_ caused decreased DNA methylation in rat offspring, nephrotoxicity, apoptosis, and necrosis of mouse kidney cells, resulting in renal oxidative damage, mitochondrial damage, and inflammatory response.

Quail, a representative animal of avians, is usually used in toxicology tests and is a common experimental animal model for evaluating the safety of mycotoxins (Jaspers, 2015). FB_1_ can enter and accumulate in animal’s body through food intake, posing a potential health threat (Chen et al., 2021; Laurain et al., 2021; Zhu and Wang, 2022). Currently, there is a lack of effective treatment for FB_1_. As such, exploring its countermeasures is urgent and important. Herein, quail was used as the experimental animal to comprehensively explore the protective role of allicin against kidney damage caused by FB_1_. The findings of this study provide an experimental basis and theoretical support for the prevention and mitigation of FB_1_ pollution.

## Materials and methods

### Animals

The Institutional Animal Care and Use Committee of Foshan University approved this study. A total of 270 healthy and active one-day-old female, yellow-feathered quails were selected for this experiment. The initial weight of the quail was 15.3 ± 0.03 g. Allicin (purchased from Shanghai Yuanye Biotechnology Co., Ltd.) feeds containing 50 mg/kg, 100 mg/kg, 500 mg/kg, and 1000 mg/kg, and FB_1_ (purchased from Shanghai Yuanye Biotechnology Co., Ltd.) feeds (30mg/kg) were obtained from the Guangdong Medical Experimental Animal Center. Allicin treatment: Firstly, allicin was weighed and then mixed into the feed to achieve allicin concentrations of 50mg/kg, 100mg/kg, 500mg/kg, and 1000mg/kg, respectively. The remaining feed was added, and the mixture was blended in a drum mixer. FB_1_ contamination treatment: The FB_1_ standard was prepared into a sterile aqueous solution at a concentration of 5mg/mL. The solution was sprayed onto the feed surface at a dosage of 30mg/kg. After manual agitation, the feed was allowed to stand for 20 min, then transferred to a mixer for blending. In the combined treatment group, allicin (500mg/kg) was first incorporated into the feed, followed by the addition of the FB_1_ solution, to ensure uniform distribution of the agents. The control feed remained untreated.

### Experimental model design

The quails were maintained ad libitum in a stable environment with 50±15 % humidity, 36±1°C, and 12h/12 h light/dark cycle for one week.

The quails were then randomly divided into 5 groups, each with 30 quails. The 5 groups were designated as groups A, B, C, D, and E. Control group A was fed with basic feed without allicin, while the feed for groups B, C, D, and E contained 50 mg/kg, 100 mg/kg, 500 mg/kg, and 1000 mg/kg of allicin, respectively.

After 8 weeks of feeding, on day 42, quails were anesthetized via ether inhalation (Han et al., 2023). Prior to anesthesia, quails were fasted for 12 hours with ad libitum access to water. Anesthesia induction was performed by placing quails in an anesthesia chamber with 4 % ether, followed by maintenance at 2 % ether concentration. Once corneal reflexes disappeared, quails were supine-fixed on a 37°C thermostatic table. Cardiac blood sampling was conducted using a 25 G needle, with 1 mL of blood collected into centrifuge tubes containing sodium heparin (10 U/mL). Tubes were inverted for thorough mixing, then centrifuged at 3000 rpm for 15 minutes. Post-blood collection, quails were euthanized with carbon dioxide. The abdominal cavity was subsequently opened, and intact kidneys were carefully excised, rinsed in ice-cold physiological saline, transferred into EP tubes, and stored at −80°C for subsequent analyses.

Another 120 quails were randomly divided into 4 groups, each with 30 quails. The groups were designated as the control group, allicin group, FB_1_ (30 mg/kg) group, and allicin+FB_1_ (30 mg/kg) group and were fed on their respective feed for 8 weeks after which the quails were subjected to fasting for 12 h, anesthetized, and their blood and kidneys were harvested on the 42nd day. The serum and kidney tissues were stored at −80°C, awaiting subsequent experiments. A portion of the kidney tissue was fixed with paraformaldehyde and used to prepare pathological sections. The remaining portion was immersed in an electron microscope fixative prepared earlier and used to prepare electron microscope sections. The contents of kidney indicators urea (**UREA**), creatinine (**CREA**), uric acid **(UA**), and urea nitrogen (**BUN**) activity in the serum were detected using a fully automatic biochemical detector. The EP tubes containing blood were centrifuged at 3000 rpm for 5 min at 2-8 °C. A total of 100 μL of the upper plasma layer was collected and assayed for UREA, CREA, UA and BUN using a fully automated biochemical analyser and accompanying enzyme kit to measure renal metabolic function in quail.

### Histopathological evaluation

The kidney tissue was fixed in paraformaldehyde for 12 hours, and then fixed in a 4°C refrigerator for another 48 hours; the tissue blocks in the embedding box were rinsed with tap water to remove the fixative; dehydrated in gradient concentrations of ethanol; transparentized in xylene twice, 10 minutes each time; immersed in wax in a 60°C constant temperature box for 2 hours, and cooled with cold water after the wax liquid solidified; the embedded wax blocks were trimmed, usually with a slice thickness of 4um; the wax slices were placed on a slide with glycerol drops and dried in a 45 °C incubator for 2 hours. After HE and Masson staining, the HE and Masson-stained sections were observed by an integrated microscope. The hematoxylin-eosin staining (H&E) stained, and Masson-stained sections were observed by microscopy to evaluate the histopathological changes in the collected pathological images. A total of 60 renal tissue sections were evaluated (5 quails per group, 3 slides per kidney). According to the following criteria(Table 1), semi-quantitative scoring of renal interstitial fibrosis, tubular lesions, and glomerular lesions was performed in accordance with reference literature(Ma et al., 2018; Xiao et al., 2018): Three non-overlapping visual fields were randomly selected for observation under a light microscope at × 400 magnification, with pathological changes scored. Three researchers who were unaware of the grouping then analyzed these pathological slides.

**Table 1:** Histopathological scoring criteria for renal tissues.

terms		Scores
Renal Tubular Vacuolar Degeneration		0: No injury
		1: <20 % vacuolar degeneration
		2: 20 %–50 % vacuolar degeneration
		3: >50 % vacuolar degeneration
Glomeruli	Structural Disarray of Glomeruli	0: No injury
		1: <20 % structural disarray
		2: 20 %–50 % structural disarray
		3: >50 % structural disarray
	Glomerular Fibrosis	0: No injury
		1: <20 % glomerular fibrosis
		2: 20 %–50 % glomerular fibrosis
		3: >50 % glomerular fibrosis
Renal Interstitial Fibrosis		0: No injury
		1: <20 % renal tissue with interstitial fibrosis
		2: 20 %–50 % renal tissue with interstitial fibrosis
		3: >50 % renal tissue with interstitial fibrosis

Kidney tissue fixed with glutaraldehyde fixative solution was immersed and washed with phosphate buffer (pH=7.2). Subsequently, the kidney was fixed with 1 % osmium acid for 1 h, dehydrated in gradient concentrations of ethanol, and embedded in epoxy resin. The samples were sliced into 55 nm and stained using the double staining method of uranyl acetate-lead citrate. A transmission electron microscope (TEM, HITACHIH-7650, Japan) was used to observe the samples and capture images.

### Quantitative real-time PCR assay

Total RNA was extracted from kidney samples using the TransZolUpPlusRNAKit® (TransGenBiotech) according to the manufacturer’s instructions. The extracted RNA was resuspended in 100 μl of RNase-free water. The concentration and purity of RNA were determined by spectrophotometry at 260/280 nm. The first-strand complementary DNA (cDNA) was then synthesized from the total RNA using a reverse transcription kit and stored at −80°C for further use. Primers are shown in Table 2. qTOWER3G system was used for qPCR analysis. Data were analyzed bydelta–deltaCt (2^−∆∆Ct^) method.

**Table 2:** qRT-PCR Primer sequences.

Type	Gene	Serial number	Primer sequences (5′−3′)	number of bases (bp)
Internal Reference	β-actin	XM_015876619.1	F:GCCCTGGCACCTAGCACAATG	21
			R:CTCCTGCTTGCTGATCCACATCTG	22
Fibrosis factor	MMP2	NM_204420.3	F:GCAGGTCGCAATGATGGCAAG	21
			R:GCAGCAACCAAGAAGAGACTGTATC	23
	TIMP2	XM_046929246.1	F:CATCAAGCGAATCCAGTACGAAGTG	23
			R:CTCCTTCTTGCCTCCTGTGTCC	21
	TIMP3	NM_205487.3	F:GCTTCCTCGCCGTGTTCCTG	20
			R:CGTCCTGCGGGTGGATGGG	20
	α-SMA	XM_015866808.1	F:GGGATGGAATCTGCTGGCAT	20
			R:GCCCGGGTACATTGTAGTGC	20
	FN-1	XM_015867978.1	F:GTGGCGAAGAAGACACTGCTGAG	22
			R:AGTTGACGGTAAGGCTGGTAGGAG	22
	PDGF	XM_015860984.2	F:AGGAGGAAGTGCGGCTGTATAG	22
			R:ACCACAACGCTTAACCAGAAGAC	23
	TGF-β1	XM_015850545.1	F:CCGACTACTGCTTCGGGACT	20
			R:TACTGTGTGTCTGCGCTCCA	20
NF-κB signaling pathway	TNF-α	MF000729.1	F:AGCTGGCGAAGACGGTGGTC	20
			R:TTCGCTGTTAGGTGACGCTGAATG	22
	NF-κB	NM_205134.2	F:GCTTCATCCGCCGCCACATC	20
			R:TCCATGACCTGCGAGCCATACC	21
	IL-1β	XM_015882931.1	F:TTACCTCCATTCTCGTGCAT	20
			R:CAGCCTTATCAAGCAACTCCA	21
	IKKα	XM_015866173.1	F:GCTGAAGGCATGGAAGGAGATGG	22
			R:CGGTCTTGGCTCTGAACTGTCTG	21
	IKKβ	XM_015883093.1	F:GTTGCCTTGCGACCTCATCCG	20
			R:ATTCATCATGGCTGCTCGCTGTC	21
Mitochondrial damage factor	COX-2	XM_015869891.2	F:TTGCGTGTTTATCAACCAGT	20
			R:CTTTGTTCACTTCGGTCCCTT	21
	COA6	XM_05157845.1	F:CTATGACAAGAACAGCATCCGAGACT	26
			R:CCCCAAAGATGTCATTCACC	20
	CYCS	XM_051281453.1	F:GACTCCATGAACAACCCCAA	20
			R:AAATCTACTCTGCCCGTGTG	20
	SIRT1	XM_051866378.1	F:ATGCTCGTTTCAGTGCCTTCGT	21
			R:GTGTCAAAGCCTGCCCCAA	20
	SIRT3	XM_015863373.1	F:CGGAGACAACAAGCACCACCA	21
			R:TTCGTGATCGTCCTACTACCC	21
	TFAM	XM_015866188.1	F:GAACCCTGCTTACATCCGAGA	21
			R:CATGAACAGGAACGCCCAT	20
	VADC-1	XM_025154858	F:TGCCAGGGATGTCTTCACCAA	21
			R:TGAGCTCGTAAATTCCAGTCCA	22
CYP450s	CYP1A1	NM_205147.2	F:TTGCGTGTTTATCAACCAGT	20
			R:CTTTGTTCACTTCGGTCCCTT	20
	CYP1A5	NM_001323211.1	F:CTATGACAAGAACAGCATCCGAGACT	26
			R:CCCCAAAGATGTCATTCACC	20
	CYP1B1	XM_419515.4	F:TTACCTCCATTCTCGTGCAT	20
			R:CAGCCTTATCAAGCAACTCCA	21
	CYP2D6	NM_001195557.1	F:GAACCCTGCTTACATCCGAGA	21
			R:CATGAACAGGAACGCCCAT	20
	AHR	NM_001323184.1	F:TTCAGGAAAGCAGAACAGCAA	21
			R:TCACAACTAATACGAAGCCAT	21
	CAR	XM_040652309.2	F:ACTTCACCTGCCCCTTTGCC	20
			R:CCTTCCTCATCCCCACGTCCA	21

*Abbreviation*: β-actin: Beta-Actin; MMP2: Matrix Metallopeptidase 2; TIMP2: Tissue Inhibitor of Metalloproteinases 2; TIMP3: Tissue Inhibitor of Metalloproteinases 3; α-SMA: Alpha-Smooth Muscle Actin; FN-1: Fibronectin 1; PDGF: Platelet-Derived Growth Factor; TGF-β1: Transforming Growth Factor Beta 1; TNF-α: Tumor Necrosis Factor Alpha; NF-κB: Nuclear Factor Kappa B; IL-1β:Interleukin 1 Beta; IKK-α/β: IκB Kinase Alpha/Beta; COX-2: Cyclooxygenase 2; COA6: Cytochrome c Oxidase Assembly Factor 6; CYCS: Cytochrome C, Somatic; SIRT1: Sirtuin 1; SIRT3: Sirtuin 3; TFAM: Transcription Factor A, Mitochondrial; VADC-1: Voltage-Dependent Anion Channel 1; CYP1A1: Cytochrome P450 1A1; CYP1A5: Cytochrome P450 1A5; CYP1B1: Cytochrome P450 1B1; CYP2D6: Cytochrome P450 2D6; AHR: Aryl Hydrocarbon Receptor; CAR: Constitutive Androstane Receptor.

### Statistical analysis

All data were analyzed using GraphPad Prism 8.0 software. Tukey’s HSD test (Benjamini et al., 2002) was employed to perform multiple comparisons to control the Type I error rate. The variances in the effect of different treatments on the nephrotoxicity of FB_1_ were determined using a one-way analysis of variance. The experimental data are expressed as means ± standard deviation (S.D.). The level of statistical significance was set at *p* < 0.05.

The heatmap was chosen due to its effectiveness in visualizing a correlation matrix (Gu, 2022). It uses color - coding (e.g., red for positive correlations, lighter shades for weaker or negative correlations) to quickly convey the strength and direction of relationships between variables. This visualization can interpret multiple correlations simultaneously. Additionally, the integration of significance symbols (*<0.05,**<0.01) complements the color scheme, allowing researchers to rapidly identify statistically significant associations.

## Results

### Selection of the optimal concentration of allicin added to quail diets

**Fig. 1:**
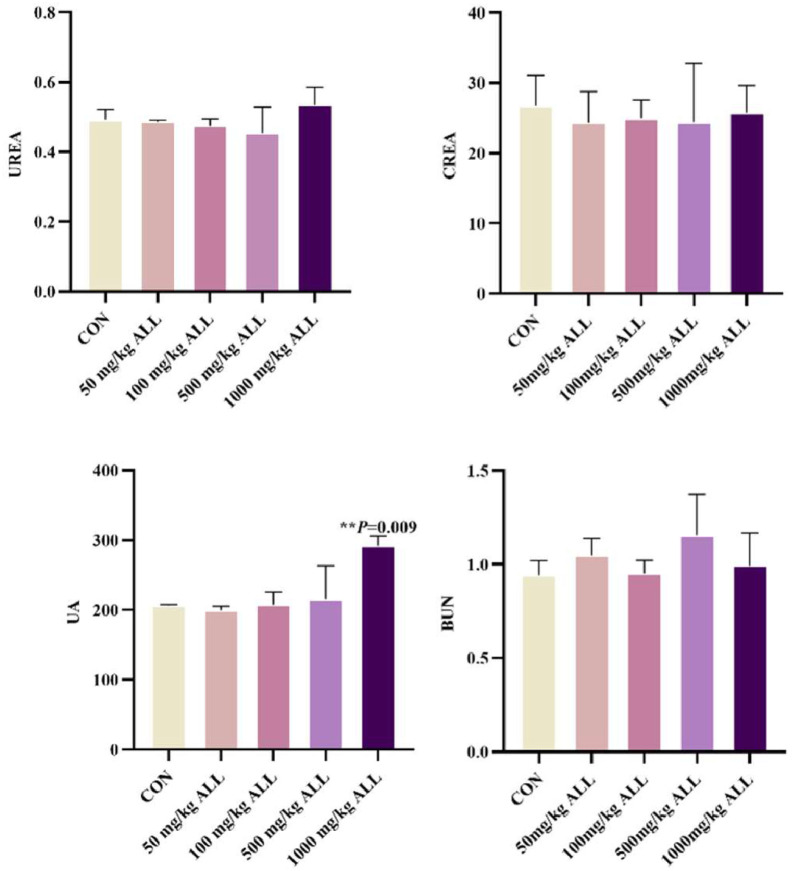
Effect of different allicin concentrations on renal biochemical indexes of quail(*n* = 5/group). Data are presented as the mean ± SD. Compared with the control group, *P < 0.05, **P < 0.01.

**Fig. 2:**
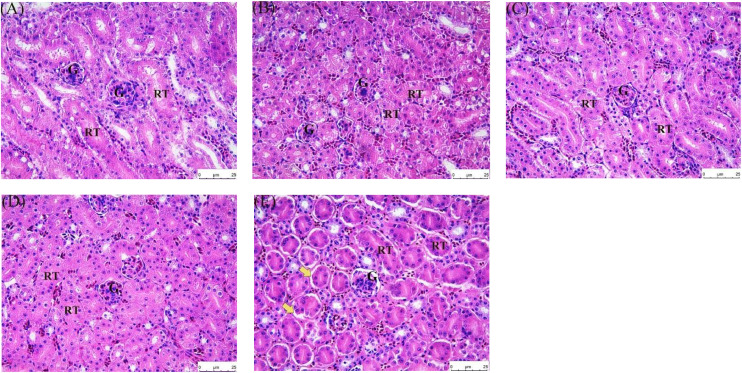
Pathological sections of HE-stained quail kidneys under different allicin concentrations. Hematoxylin and eosin stains (200 ×). (A)Control group;(B)50mg/kg ALL group;(C)100mg/kg ALL group;(D)500mg/kg ALL group;(E)1000mg/kg ALL group. G: Glomerulus, RT: Renal Tubule. Yellow arrows represent vacuolar degeneration. Scale bar: 25 μm.

Renal biochemical markers and HE staining were used to detect changes in the kidneys of quail after treatment with different concentrations (50 mg/kg, 100 mg/kg, 500 mg/kg, 1000 mg/kg) to explore the optimal concentration of allicin in quail diet. As depicted in the Fig. 1, there were no statistically significant differences in the levels of UREA, CREA, and BUA between the control group and the groups treated with different doses of allicin (*P* > 0.05). However, regarding the UA index, the uric acid level in the group treated with 1000 mg/kg ALL approached 300 μmol/L, which was significantly higher than that in the control group (*P* < 0.05). Kidneys of quails in groups A, B, C, and D exhibited ideal histological characteristics after HE stains. The morphology of glomeruli and tubules was intact, the cell membrane structure was intact, and nuclei staining was uniform and in a normal state (Fig. 2 and Table 3). Noteworthy, the gradual increase in drug dose enhanced the morphological structure between cells and generally increased the number of cells, which suggested a positive correlation between drug dose and cell health within a certain range. However, the kidneys of quails in group E exhibited abnormal histological changes, such as significant enlargement of intercellular space and intercellular hemorrhage, with a pathological score of 1.6 ± 0.48, suggesting that the treatment conditions in this group were unfavorable to the cells. The optimal dosage of allicin was taken to be 500mg/kg after consideration of various factors. This allicin concentration was used in subsequent experiments.

**Table 3:** Histopathological section scoring of quail kidney. Data are presented as the mean ± SD.

Groups	Score
Control group	0 ± 0
50mg/kg ALL group	0.5 ± 0.7
100mg/kg ALL group	0.66 ± 0.96
500mg/kg ALL group	0.33 ± 0.46
1000mg/kg ALL group	1.6 ± 0.48

### Allicin attenuates FB_1_-induced renal pathological damage

**Fig. 3:**
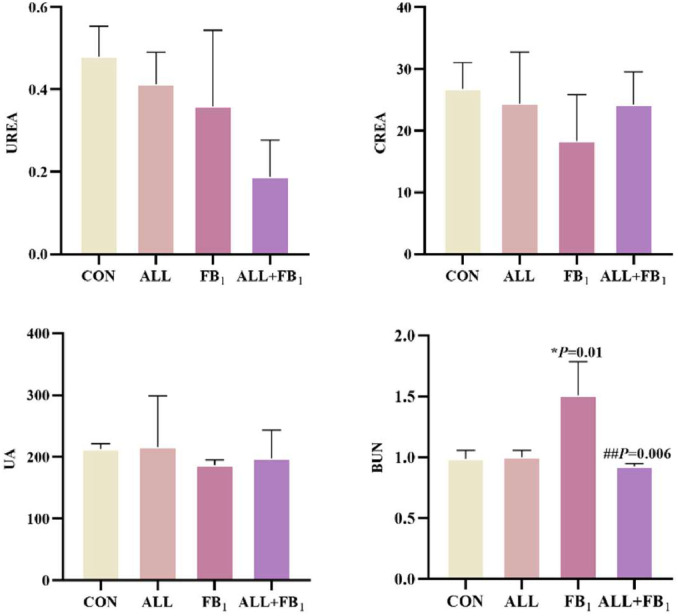
Effect of allicin on biochemical markers of renal injury in quail caused by FB_1_(*n* = 5/group). Data are presented as the mean ± SD. Compared with the control group, *P < 0.05, **P < 0.01; Compared to the FB_1_ (30mg/kg) group, #*P* < 0.05, ##*P* < 0.01.

Changes in biochemical indexes, HE stains, and transmission electron microscopy were observed and used to determine the effect of allicin on FB_1_-induced quail kidney. As shown in Fig. 3, compared with the control group, there were no significant differences in the levels of UREA, CREA, and UA in the FB_1_ group, ALL group, and ALL+FB_1_​ group (*P* > 0.05). The BUN level in the FB_1_ group was significantly higher than that in the control group (*P* < 0.05), while there was no significant difference in the BUN level in the ALL group (*P* > 0.05). The BUN level in the ALL+FB_1_​ group was significantly lower than that in the FB_1_ group and showed no significant difference compared with the control group (*P* > 0.05).

**Fig. 4:**
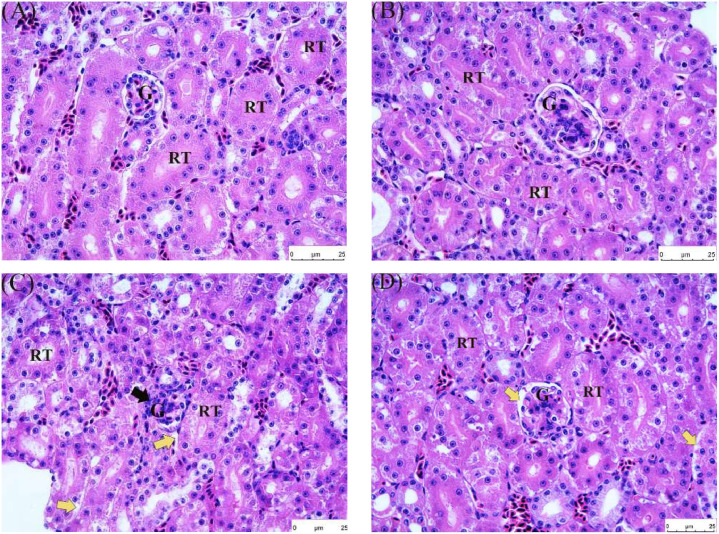
Pathological changes of kidney injury induced by FB_1_ and treated with allicin. Hematoxylin and eosin stains (200 ×). (A) Control group; (B) Allicin (500mg/kg) group; (C) FB_1_ (30mg/kg) group; (D) 30mg/kg FB_1_+500mg/kg allicin group. G: Glomerulus, RT: Renal Tubule. Black arrows represent glomerular structural disorders, and yellow arrows represent vacuolar degeneration. Scale bar: 25 μm.

The pathological sections of quail kidney tissues were stained by HE as illustrated in Fig. 4. The results are as follows (Table 4):

**Table 4:** Histopathological Section Scoring of Quail Kidney. Data are presented as the mean ± SD.

Groups	Score
Control group	0 ± 0
500 mg/kg ALL group	0.33 ± 0.46
30 mg/kg FB_1_ group	2 ± 0.82
30 mg/kgFB_1_+500 mg/kg allicin group	1.3 ± 0.47

Group A: Glomeruli and renal tubules displayed relatively distinct structures, with cells arranged in an orderly manner and no obvious morphological abnormalities. The interstation showed no signs of inflammatory cell infiltration, hemorrhage, or fibrosis. Group B: Glomerular and tubular morphology remained largely normal, with relatively clear cellular boundaries comparable to the control group. The overall renal tissue architecture was well-maintained, featuring distinct cellular layers and an interstation without significant widening or abnormal substance deposition. Group C: In contrast, glomeruli exhibited structural disarray (black arrows), with renal tubular cells arranged irregularly, narrowed luminal spaces, and detachment of tubular epithelial cells accompanied by vacuolization (yellow arrows). Group D: Glomerular and tubular structures were mostly preserved, with no severe cellular deformation. However, compared to Groups A and B, cellular arrangement appeared slightly disordered, with mild widening of intercellular spaces in focal areas and subtle vacuolar degeneration in renal tubules.

**Fig. 5:**
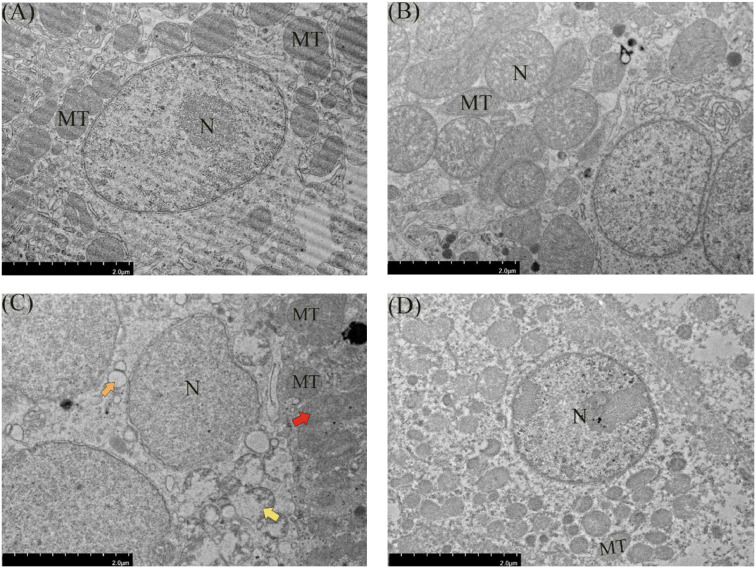
Ultrastructural changes of quail kidneys induced by FB_1_ and treated with allicin. Transmission electron microscopy (5000 ×). (A) Control group; (B) Allicin (500mg/kg) group; (C) FB_1_ (30mg/kg) group; (D) 30mg/kg FB_1_+500mg/kg allicin group. Note: MT: mitochondria; N: nucleus. Red arrow represents mitochondrial swelling, and orange arrow represents vacuolation; The yellow arrow represents nuclear fragmentation. Scale bar:2 μm.

The structure of renal tubular epithelial cells in groups A and B was complete, the cytoplasm was evenly distributed, and the structure was normal. The mitochondrial structure of renal tubular epithelial cells was normal with complete morphology, and the mitochondrial cristae were closely arranged (Fig. 5). The edges of renal tubular epithelial cells in group C were blurred and vacuolized, and the mitochondria of renal tubular epithelial cells were swollen, sunken, and distorted. In group D, the structure of renal tubular epithelial cells was intact, the cytoplasm was evenly distributed, and the structure of mitochondria of renal tubular epithelial cells was normal.

### Allicin inhibits FB_1_-induced mRNA expression of heteronuclear receptors and that of related genes in kidneys

**Fig. 6:**
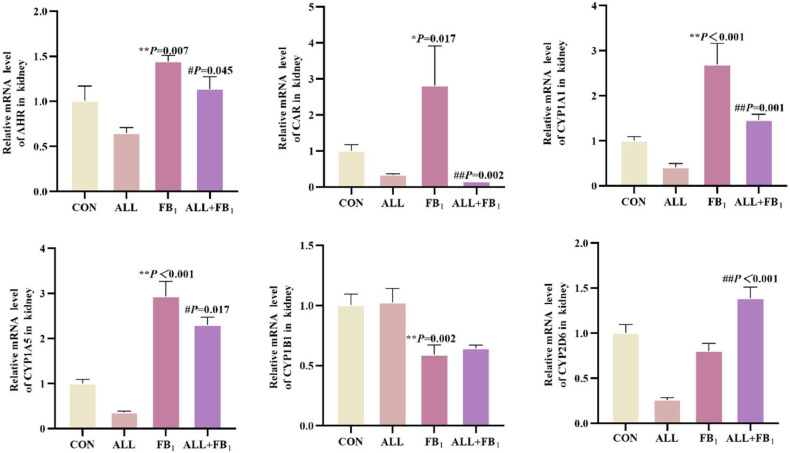
The mRNA expression of heterologous nuclear receptors and related subunits in quail kidney tissues under different treatments(*n* = 5/group). Data are presented as the mean ± SD. Compared with the control group, *P < 0.05, ^⁎⁎^*P* < 0.01; Compared to the FB_1_ (30mg/kg) group, ^#^*P* < 0.05, ^##^*P* < 0.01.

The NXR response and CYP450 enzyme system content were determined and analyzed to assess the effect of allicin on FB_1_-induced kidney CYP450s in quail (Fig. 6). Compared to the control group (*P < 0.05, **P < 0.01), FB_1_ significantly increased respectively the mRNA expression of AHR (+45 %), CAR (+182 %), CYP1A1 (+170 %), and CYP1A5 (+193 %); FB_1_ significantly decreased the mRNA expression of CYP1B1 (−60 %), CYP2D6 (−20 %). In addition, Allicin treatment significantly reduced the mRNA expression of −22 %, −94 %, −14 %, −78 %, −21 %, 73 %respectively compared to FB_1_ group(^#^*P* < 0.05, ^##^*P* < 0.01). These results suggested that exposure to FB_1_ disrupts NXR response and CYP450 enzyme system, while allicin inhibits FB_1_-induced NXR response and CYP450 enzyme system.

### Allicin inhibits FB_1_-induced renal fibrosis

Masson staining was performed to detect the expression of fibrosis-related genes to verify the correlation between fibrosis and allicin on FB_1_-induced quail kidney damage.

**Fig. 7:**
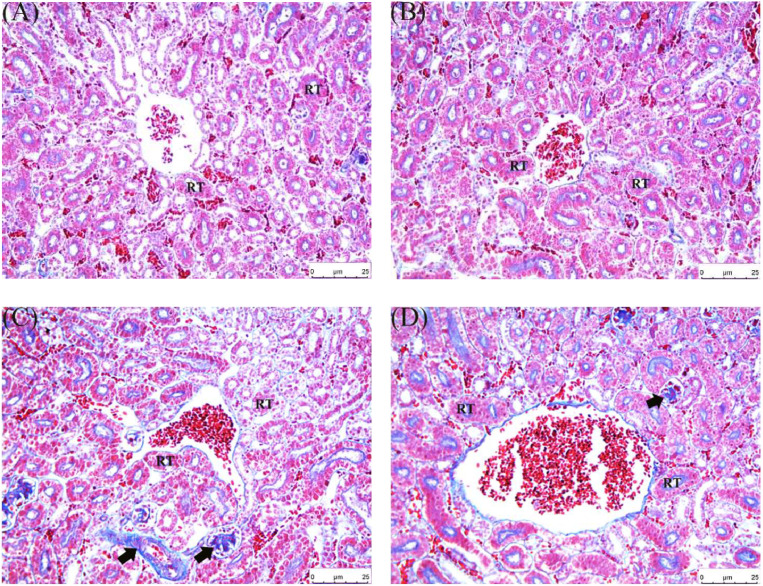
FB_1_-induced pathological changes causing kidney fibrosis in quail. Masson trichrome stain (200 ×). (A) Control group; (B) 500mg/kg allicin group; (C) 30mg/kg FB_1_ group; (D) Treatment group. RT: Renal Tubule. Black arrows indicate the presence of fibrosis. Scale bar: 25 μm.

Notably, collagen fibers in groups A and B were insignificant, and the structure of the renal tubules was clear (Fig. 7). In contrast, collagen fibers in group C significantly increased, there was tubulointerstitial fibrosis, the glomerular structure was disordered, and the cell arrangement was irregular and loose. Group D had a reduction in collagen fibers, the cells were more neatly arranged, and the structure was clear.

**Fig. 8:**
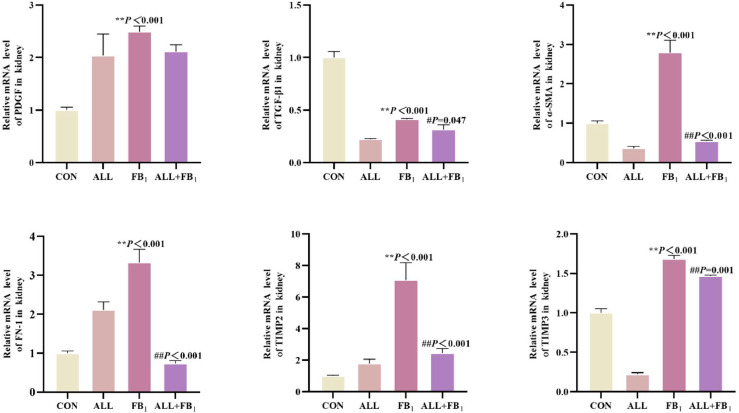
The mRNA expression of cytokines associated with renal fibrosis(*n* = 5/group). Data are presented as the mean ± SD. Compared with the control group, *P < 0.05, ^⁎⁎^*P* < 0.01; Compared to the FB_1_ (30mg/kg) group, ^#^*P* < 0.05, ^##^*P* < 0.01.

As shown in Fig. 8, compared to the control group (*P < 0.05, **P < 0.01), FB_1_ significantly increased respectively the mRNA expression of PDGF (+149 %), α-SMA (+179 %), FN-1 (+232 %), TIMP2 (+609 %) and TIMP3 (+68 %); FB_1_ significantly decreased the mRNA expression of TGF-β1 (−59 %). In addition, Allicin treatment significantly reduced the mRNA expression of −15 %, −80 %, −78 %, −65 %, −13 %, −24 % respectively compared to FB_1_ group(#*P* < 0.05, ##*P* < 0.01). These results suggested that allicin inhibits FB_1_-induced increase in renal fibrosis.

### Allicin inhibits the mRNA expression of the NF-κB signaling pathway caused by FB_1_

**Fig. 9:**
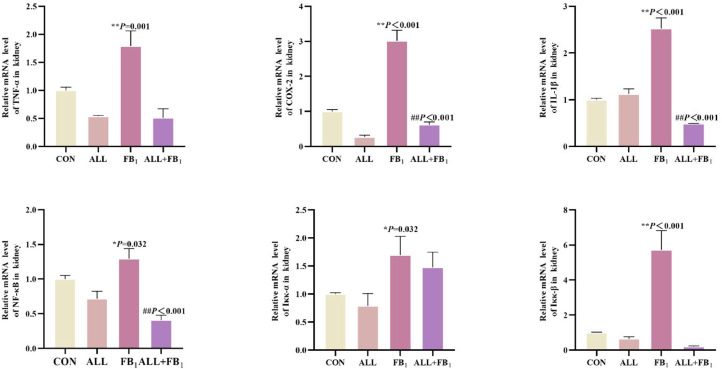
The mRNA expression of cytokines associated with kidney inflammation(*n* = 5/group). Data are presented as the mean ± SD. Compared with the control group, *P < 0.05, ^⁎⁎^*P* < 0.01; Compared to the FB_1_ (30mg/kg) group, ^#^*P* < 0.05, ^##^*P* < 0.01.

Genes associated with the NF-κB pathway were examined to determine whether allicin plays a role in the anti-inflammatory mechanism of FB_1_-induced kidney inflammation (Fig. 9). Compared to the control group (*P < 0.05, **P < 0.01), FB_1_ significantly increased respectively the mRNA expression of TNF-α (+79 %), COX-2(+201 %), NF-κB (+29 %), Iκκ-α (+68 %), Iκκ-β (+473 %), and IL-1β (+169 %). In addition, Allicin treatment significantly reduced the mRNA expression of −72 %, −79 %, −68 %, −13 %, −82 % respectively compared to FB_1_ group(#*P* < 0.05, ##*P* < 0.01). These results highlighted that allicin inhibits the expression of NF-κB pathway-related genes induced by FB_1_.

### Allicin inhibits FB_1_-induced renal mitochondrial damage

**Fig. 10:**
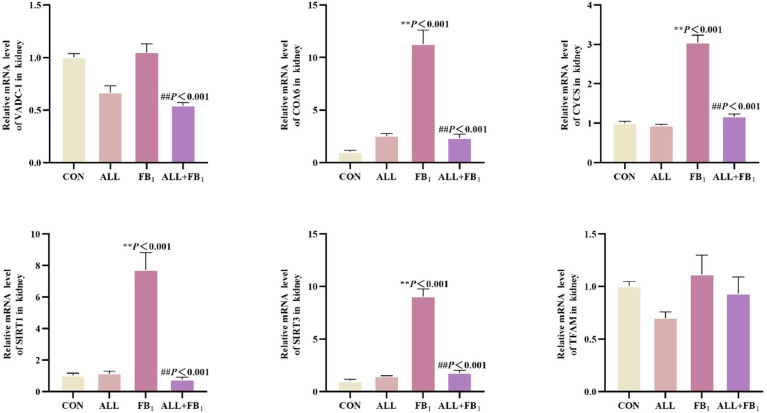
The mRNA expression of cytokines associated with kidney mitochondrial damage(*n* = 5/group). Data are presented as the mean ± SD. Compared with the control group, *P < 0.05, ^⁎⁎^*P* < 0.01; Compared to the FB_1_ (30mg/kg) group, ^#^*P* < 0.05, ^##^*P* < 0.01.

The mRNA expression of the mitochondrial damage-related gene was determined to confirm the effect of allicin on FB_1_-induced kidney mitochondrial damage(Fig. 10). Compared to the control group (*P < 0.05, **P < 0.01), FB_1_ significantly increased respectively the mRNA expression of VADC-1 (+5 %), COA6 (+1028 %), CYCS (+205 %), SIRT1 (+672 %), SIRT3 (+804 %). In addition, Allicin treatment significantly reduced the mRNA expression of −49 %, −79 %, −62 %, −90 %, −80 %respectively compared to FB_1_ group(#*P* < 0.05, ##*P* < 0.01).These results indicated that allicin significantly inhibits FB_1_-induced mitochondrial damage. This finding was consistent with mitochondrial damage observed using transmission electron microscopy.

### Correlation analysis of CYP450 enzyme system, fibrosis, NF-κB signaling pathway and mitochondrial damage factors

**Fig. 11:**
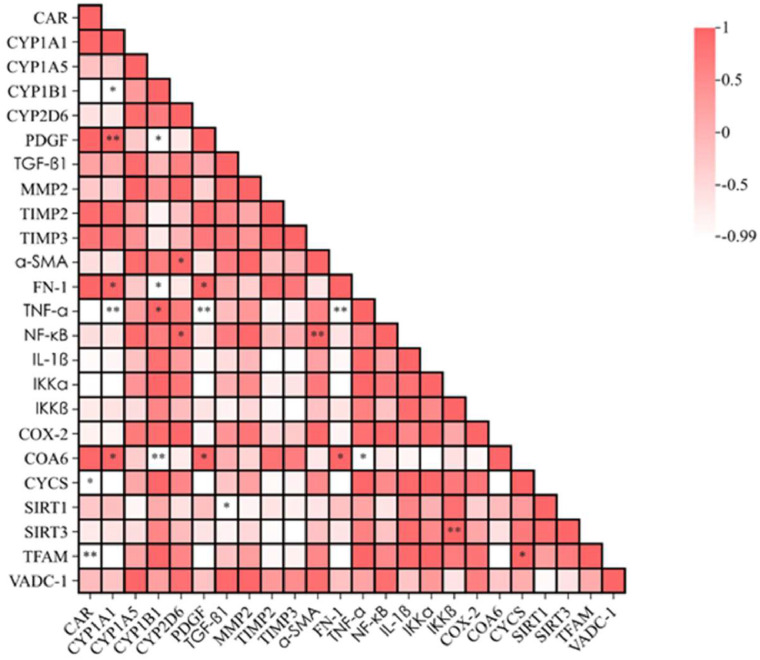
Correlation analysis of mRNA expression levels related to CYP450 enzyme factor, NF-κB inflammatory pathway, fibrosis factor and mitochondrial damage factor. Compared with the control group, *p < 0.05, ^⁎⁎^*p* < 0.01.

Pearson correlation analysis was done to investigate the relationship between the CYP450 enzyme system, fibrosis, NF-κB signaling pathway, and mitochondrial damage factors. A correlation coefficient close to 1 denoted a strong positive correlation, while a correlation coefficient close to −1 denoted a strong negative correlation. A correlation coefficient close to 0 denoted weak or no correlation. Fig. 11 shows the results of kidney-related analysis. CAR expression was negatively correlated with levels of mitochondrial damage factors CYCS and TFAM. CYP1B1 expression was positively correlated with the level of inflammatory TNF-α, but negatively correlated with the levels of CYP1A1, fibrosis factor PDGF, FN-1, and mitochondrial damage factor COA6. The expression of CYP1A1 was positively correlated with the levels of fibrosis factor PDGF, FN-1, and mitochondrial damage factor COA6. CYP2D6 expression was positively correlated with levels of NF-κB and fibrosis factor α-SMA in the NF-κB signaling pathway. The expression of NF-κB in the NF-κB signaling pathway was positively correlated with the level of α-SMA. Iκκ-β expression in the NF-κB signaling pathway was positively correlated with the level of mitochondrial damage factor SIRT3. Inflammatory cytokine TNF-α was negatively correlated with the levels of CYP1A1, PDGF, and FN-1. The expression of fibrosis factor FN-1 was positively correlated with levels of fibrosis factor PDGF and mitochondrial damage factor COA6. PDGF was positively correlated with the level of mitochondrial damage factor COA6, while TGF-β1 was negatively correlated with the level of mitochondrial damage factor SIRT1. The expression of mitochondrial damage factor TFAM was positively correlated with the level of CYCS.

## Discussion

In warm and humid South China, mycotoxins (typically represented by FB_1_) can significantly damage the immune system, induce oxidative stress and inflammatory responses, and cause serious harm and adverse effects on human and animal health (Dai et al., 2024; Zhang et al., 2023). FB_1_ is difficult to effectively remove through conventional physical or chemical means during the growth, planting, harvesting, storage, and processing of grains because of its unique physical structure and complex biological characteristics (Hoffmans et al., 2022). As such, it continues to remain in grains and related products (Bryła et al., 2017). Herein, a series of exploratory research was carried out to find suitable feed additives and screen their optimal concentrations to effectively alleviate toxic reactions caused by FB_1_. This study focused on allicin by exploring its potential protective effects against FB_1_ because of its antioxidant and anti-inflammatory properties. The study analyzed the molecular mechanism of allicin's protective effect in alleviating kidney damage caused by FB_1_ with an aim of providing a solid theoretical foundation and scientific basis for subsequent in-depth research and practical application in other relevant fields.

Quail is an excellent indicator organism in monitoring environmental pollution and is widely used in studies related to stress response physiology (Laurence et al., 2015). To date, investigations into allicin’s role in alleviating FB_1_-induced quail nephrotoxicity remain relatively limited. It is thus urgent to further explore the internal mechanisms of FB_1_-induced renal toxicity in quail and identify effective ways to alleviate renal damage caused by FB_1_. In this study, different concentrations of allicin were added to the quail diet, after which renal biochemical indicators were observed to determine whether there were any changes. Notably, compared with the control group, there were no statistically significant differences in the levels of UREA, CREA, and BUA between the control group and the groups treated with different doses of allicin (*P* > 0.05). However, regarding the UA index, the uric acid level in the group treated with 1000 mg/kg ALL approached 300 μmol/L, which was significantly higher than that in the control group (*P* < 0.05). This suggests that high dose allicin may disrupt the uric acid metabolic processes in quails, and the elevation in uric acid levels may be associated with alterations in renal excretory function or intrabody purine metabolic pathways. The underlying mechanisms warrant further investigation. The kidney pathological sections of quails in groups A, B, C, and D had relatively ideal histological characteristics, with an intact morphology of glomeruli and renal tubules, intact cell membrane structure, and uniform and normal staining of cell nuclei. Noteworthy, the gradual increase of drug dose enhanced the morphological structure between cells and generally increased the number of cells, indicating a positive correlation between drug dose and the good state of cells within a certain dose range. However, pathological sections of quails in group E exhibited abnormal histological changes, with significantly enlarged intercellular spaces and intercellular bleeding, suggesting that the allicin dose used for this group had a negative effect on cells. Therefore, an allicin dose of 500 mg/kg was used for the subsequent experiments. A quail kidney injury model induced by FB_1_ was then established. Subsequent pathological analysis revealed disarrangement of cells in the renal tissue and significant vacuolation accompanied by fibrosis. Transmission electron microscopy further revealed pathological changes, such as mitochondrial swelling, vacuolar degeneration, and nuclear fragmentation. These findings strongly demonstrate that FB_1_ can induce significant renal damage in quail (Guerre et al., 2022; Kövesi et al., 2024; Xu et al., 2021). Noteworthy, the pathological damage of the kidney microstructure was effectively alleviated by allicin treatment (Arellano Buendia et al., 2023; El-Kashef et al., 2015). Herein, Besides the significant fluctuations in the BUN parameter, no remarkable changes were noted in the remaining serum biochemical indicators of quail kidneys. This suggests that FB_1_ can elevate the BUN level in quails, possibly impairing the kidney’s handling capacity for nitrogenous metabolic products. When allicin is co-treated with FB_1_, allicin can effectively reduce the elevation of BUN level induced by FB_1_, restoring it to a level close to the normal one. In contrast, previous studies postulate that exposure to FB_1_ toxins can cause abnormal serum index levels in quail and other poultry (Li et al., 2023; Tardieu et al., 2007, 2006; Xu et al., 2021). This deviation was attributed to the fact that FB_1_ mainly interferes with the normal physiological function of cells by inhibiting the synthesis of sphingolipids, which was possibly different in the target of its action on renal cells. The renal cells of quail are possibly less sensitive to FB_1_, leading to insignificant changes in biochemical indicators.

NXRs, including AHR, CAR, and PXR, regulate the expression of various xenobiotic metabolic enzymes and transporters. Of note, their regulatory effect on CYP has attracted much attention (Gharavi and El‐Kadi et al., 2005; Hakkola et al., 2018). CYP, a vital member of xenobiotic enzymes, occupies a central position in the biotransformation, metabolism, and detoxification process of xenobiotics (Guéguen et al., 2006). Specifically, AHR belongs to the Per-Arnt-Sim (PAS) family of transcription factors and can transactivate phase I enzymes, such as CYP1A1, CYP1A2, and CYP1B1 (Chirulli et al., 2007; Gharavi and El‐Kadi, 2005). CAR and AHR synergistically regulate the expression of PXR (Gharavi and El‐Kadi, 2005; Jung et al., 2006). Previous studies postulate that FB_1_ can significantly enhance the expression of CYP in the kidneys of experimental animals (Cao et al., 2020; Kövesi et al., 2024). In contrast, allicin can inhibit the participation of NXR and CYP450 enzyme systems in metabolizing toxins in the body, thereby reducing the expression of related genes (Nan et al., 2021). In this study, the expression of AHR, CAR, CYP1A1, CYP1A5, and CYP1B1 genes in the FB_1_ group exhibited a significant upregulation trend consistent with the findings of previous studies (Kövesi et al., 2024). However, the expression of these genes was normalized by allicin treatment. This finding indicates that NXRs and CYP450 enzyme systems are involved in the metabolism of FB_1_ in vivo. The results of this study demonstrate that NXRs and CYP450 enzyme systems are involved in the metabolic pathway and physiological processes of FB_1_ in the body, while allicin effectively acts on this metabolic system because its unique biological activity, ultimately restoring the expression of the genes back to normal.

Inflammation often accompanies the occurrence and development of kidney damage. NF-κB, an inducible cytoplasmic transcription factor, occupies a core position in coordinating and inducing proinflammatory genes. It can regulate many genes involved in various inflammatory and immune processes (Oeckinghaus and Ghosh, 2009; Yu et al., 2020). Despite the great importance of the NF-κB signaling pathway in inflammatory response, studies on FB_1_-induced NF-κB signaling pathway are relatively scarce. This study aimed to evaluate whether FB_1_ exposure could activate the NF-κB signaling pathway in quail kidneys to mediate inflammation. The mRNA expressions of TNF-α and IL-1β increased significantly, which continuously activated the NF-κB signaling pathway, potentially leading to kidney inflammation. The histopathological changes, such as glomerular and tubular structural disorders, suggested the presence of kidney inflammation. These findings collectively suggested that FB_1_ can successfully induce an inflammatory response in the quail kidney by regulating the expression of NF-κB. Noteworthy, the expression of factors associated with the NF-κB signaling pathway in the FB_1_ antagonist group completely contrasted with that of the FB_1_ group, while the associated inflammatory factors exhibited a downward trend. This result is highly consistent with that of previous studies on the anti-inflammatory properties of allicin (Jin et al., 2013; Sela et al., 2004; You et al., 2019). The expression of the NF-κB signaling pathway in the FB_1_ antagonist group was thus significantly lower than that in the FB_1_ group.

A long-term inflammatory response caused by kidney damage can gradually induce renal fibrosis. Epithelial cells and endothelial cells in the renal interstitial undergo phenotypic transformation and then differentiate into fibroblasts when the body is exposed to adverse external environmental stimulation. This process has been confirmed in previous studies (Yuan et al., 2019). Fibroblasts can secrete and synthesize collagen fibers and fibronectin. However, these substances are difficult to effectively degrade by the body, leading to continuous accumulation and deposition of large amounts of extracellular matrix (ECM), especially collagen fibers, in the renal tissue. Many studies postulate that TGF-β1 promotes the proliferation activity of fibroblasts and can induce them to differentiate into myofibroblasts, thereby accelerating fibrosis (Meng et al., 2016). Previous studies have focused on the effect of FB_1_ on animal liver fibrosis, confirming that FB_1_ can induce liver fibrosis (Bondy et al., 2000; Cao et al., 2022). The results of this study revealed significant pathological characteristics of fibrosis and enhancement of transcription levels of fibrosis marker genes after exposure of quail kidneys to FB_1_. Noteworthy, FB_1_ exposure paradoxically caused a significant decrease in TGF-β1 levels, possibly because of its effects on multiple links, such as synthesis, secretion, and degradation of TGF-β1. These effects could be potentially single or synergistic. However, the specific mechanism needs further experiments to clarify. This study focused on determining the effect of allicin on the expression of FB_1_-induced renal fibrosis-related genes to further understand the mechanism by which allicin exerts its anti-fibrotic properties. The expression of fibrosis-related factors in renal tissue exhibited a significant downward trend after allicin treatment. These results highlight that allicin potentially regulates the expression of fibrosis-related factors mediated by FB_1_, thereby exerting a protective effect on quail kidneys, effectively antagonizing renal fibrosis.

Mitochondria serve as energy factories and signaling hubs within cells (Picard and Shirihai, 2022). The integrity of their functions is essential for maintaining normal physiological activities of kidney cells. Mitochondrial dysfunction is a vital pathological link in various renal diseases, including diabetic nephropathy, chronic kidney disease, and renal dysfunction (Flemming et al., 2018; Galvan et al., 2017). Previous studies postulate that FB_1_ can cause mitochondrial damage in kidney cells (Guerre et al., 2022; Voss et al., 2001). In this study, transmission electron microscopy was used to observe quail kidneys after FB_1_ exposure. Notably, typical mitochondrial damage features, such as mitochondrial swelling and vacuolation, were observed. These results confirmed the toxic effects of FB_1_ on renal mitochondria, indicating that FB_1_ can disrupt the balance of energy metabolism and signal transduction in kidney cells. Mitochondria can exert their protective effects by directly or indirectly upregulating several mitochondrial endogenous genes that contribute to the maintenance of mitochondrial function, including PRDX3, SIRT1, SIRT3 and TFAM, under mild stress conditions (Yang et al., 2018). TFAM binds to mitochondrial DNA and directly regulates mitochondrial genome transcription and replication (Scarpulla, 2008). CYCS is upregulated, prompting the release of cytochrome C from mitochondria into cells when mitochondria are dysfunctional, which triggers a series of oxidative stress responses, enhancing cellular oxidation and increasing oxygen utilization. COA6 participates in the regulation of ATP synthesis by catalyzing cytochrome oxidation reactions (Arumugam et al., 2021). Herein, the expression of CYCS and COA6 was significantly upregulated after FB_1_ exposure, indicating that mitochondrial dysfunction triggered a stress compensatory response in cells. Noteworthy, the expression of mitochondrial function-related genes was significantly decreased after allicin treatment, suggesting that allicin can effectively alleviate FB_1_-induced mitochondrial damage and restore normal mitochondrial function.

FB_1_ produced multiple toxic effects, including disturbances in CYP450 enzymes and NXRs, activation of inflammatory responses, promotion of renal fibrosis, and induction of mitochondrial damage on quail kidneys. Specifically, FB_1_ activates the NF-κB signaling pathway in the kidney, leading to a massive release of inflammatory factors, which in turn triggers an inflammatory response and significantly increases the body's inflammatory level. FB_1_ also upregulated the expression of NXRs and CYP450 enzymes, destroying the normal functioning of the CYP450 enzyme system, leading to renal metabolic disorders and abnormally elevated metabolic levels. Moreover, FB_1_ promoted the expression of fibrosis-related genes, presenting complex pathological regulatory mechanisms. FB_1_-induced mitochondrial damage manifested itself as a destruction of the mitochondrial structure and abnormal changes in the expression of function-related genes, further exacerbating renal cell damage and dysfunction. Noteworthy, Allicin exhibited a significant ability to alleviate the toxicity of FB_1_. Its mechanism of action potentially involved multiple levels, including direct inhibition of FB_1_-induced inflammatory response, regulation of fibrosis-related gene expression, repair of mitochondrial damage, and restoration of normal functioning of the CYP450 enzyme system. These results suggest that allicin has a certain effect on alleviating FB_1_ poisoning, highlighting its broad application prospect in drug research and development and feed additives. The findings of this study provide solid and reliable experimental data support for allicin use as an effective means to alleviate FB_1_ and other mycotoxin poisoning.

In this study, the protective effect of allicin on FB_1_-induced renal injury in quail was preliminarily demonstrated through dose screening and mechanism exploration. However, the present study has limitations in mechanistic exploration and model translation due to species differences. Future research could focus on: Testing in mammalian models (mice, rats) to clarify species variations; Using adult/geriatric quail models for age-related analysis; Investigating allicin's long-term safety, pharmacokinetics, and toxin interactions.

## CRediT authorship contribution statement

**Yangwan Zhang:** Writing – review & editing, Writing – original draft, Data curation, Validation, Formal analysis. **Yihao He:** Supervision, Data curation. **Xueyan Zhu:** Data curation, Supervision. **Yang Liu:** Funding acquisition, Supervision. **Changyu Cao:** Supervision, Data curation, Writing – review & editing.

## Declaration of competing interest

The authors declare that they have no known competing financial interests or personal relationships that could have appeared to influence the work reported in this paper.
